# Gender Differences Between Disability, Quality of Life, and Sedentary Behavior in Individuals with Chronic Non-Specific Neck Pain

**DOI:** 10.3390/jcm14228155

**Published:** 2025-11-17

**Authors:** Anna Christakou, Alexandra Loizou, Dimitrios Chytas

**Affiliations:** 1 Laboratory Biomechanics, Physiotherapy Department, School of Health Sciences, University of Peloponnese, 23100 Sparta, Laconia, Greece; 2 Physiotherapy Department, School of Health Sciences, University of Peloponnese, 23100 Sparta, Laconia, Greece; alex.loizou2002@gmail.com; 3 Laboratory Basic Sciences, Physiotherapy Department, School of Health Sciences, University of Peloponnese, 23100 Sparta, Laconia, Greece; dimitrios.chytas@uop.gr

**Keywords:** chronic non-specific neck pain, disability, quality of life, sedentary life, gender

## Abstract

**Introduction**: Chronic non-specific neck pain is a musculoskeletal disorder which may affect psychological well-being and work performance. Depression, anxiety, and limitations in daily and work activities may occur differently between genders in individuals with chronic non-specific pain. The main purpose of the present cross-sectional study was to investigate the differences between men and women with chronic non-specific neck pain in disability due to pain, quality of life, and sedentary behavior. Secondly, we investigated the intercorrelations between the three above measured variables in the total sample. **Methods**: Eighty patients (44 men and 36 women), aged 20–55 years (Μ = 33.55, SD = 11.16) with chronic non-specific neck pain in the last 3 years participated. They completed three validated questionnaires which measured neck disability pain, quality of life, and sedentary behavior with (a) the Neck Disability Index (NDI), (b) the quality-of-life EuroQol-5D (EQ-5D), and (c) the International Physical Activity Questionnaire (IPAQ), respectively. Gender comparisons with Kruskal–Wallis H tests and correlation analysis with Spearman r tests were performed between the above variables using SPSS 29.00. **Results**: Men reported (a) better quality of life (H = 16.14, *p* < 0.001), (b) lower pain-related disability (H = 13.96, *p* < 0.001), (c) more time spending in vigorous physical activity (H = 3.37, *p* < 0.05), (d) more time spending in moderate physical activity (H = 5.17, *p* < 0.05), and (e) more time spending in walking (H = 11.24, *p* < 0.001). A strong positive correlation was found between the NDI and the EQ-5D Index (r = 0.74, *p* = 0.002) and a negative correlation was found between NDI and the EQ-5D VAS (r = −0.65, *p* = 0.003). **Discussion**: The present findings reported that men have more time walking, and have lower disability due to neck pain than women, thus they have better quality of life than women with chronic non-specific neck pain.

## 1. Introduction

Chronic non-specific neck pain is a musculoskeletal problem which affects approximately 70% of the population at least once in their lives [1,2]. It is an unpleasant pain with varying intensity and frequency, either continuous or intermittent [3]. Its main characteristics include the absence of a specific cause, usually presenting with radiating pain in the neck and shoulder and worsening at certain joint movement angles [4]. It may cause a reduction in performing everyday activities including self-care, work, and leisure [5] which leads to disability and reduced quality of life [6].

The etiology of neck pain is complex with the onset and course of pain. The biopsychosocial model is commonly used in chronic pain and physiotherapy should integrate holistic treatment to address all components of chronic issues, such as physical, neurophysiological, psychosocial, environmental, and personal factors [7]. Particularly, in the physical domain, job-related exposure, impairments in cervicoscapular strength, mobility, and endurance may be associated with neck pain [8]. In the neurophysiological domain, studies reported alterations in pain processing that result in localized and widespread hypersensitivity to mechanical stimuli [8]. Psychosocial elements including job satisfaction and psychological state have been shown to predict long-term symptoms and disability correlated with neck pain [8].

The prevalence of neck pain, regardless of source, may be higher in females [7]. These gender differences may be a result from physiological, hormonal, or psychosocial mechanisms. In particular, in neck pain, the differences between gender may be due to muscle stiffness or alterations in neuromuscular control [9]. Females were found to be twice as likely to have persistent pain after neck trauma, likely due to higher vulnerability to tissue damage based on structural factors, higher risk for psychological distress, the pain sensitization process, and possibly social factors [10]. Anatomically, females have smaller cervical vertebrae resulting in less segmental support area (disk and facet joints), less muscle strength, and ligament stiffness resulting in increased pain, reduced stability and increased range of motion, lower tolerance limit for lower neck shear force, and faster muscle reaction times resulting in greater tissue strain and injury potential [10]. Furthermore, it is well known that sex hormones can affect pain in both sexes [11]. In men, there is a gradual decline in sex hormones with age, whereas women experience an abrupt decrease after menopause. The incidence of pain increases in women after puberty and varies through menopause [12]. In addition, testosterone in males is protective against pain, while mixed effects are noted with the female sex hormone, estrogen [13,14].

Quality of life is influenced by the individual’s physical health, psychological state, personal attitudes, social surroundings, and their relationship with their environment [15]. Sedentary lifestyle and quality of life seem to be inextricably linked concepts with regard to the psychosomatic state of the individual. Boberska et al. [16] reported that the reduced daily sedentary behavior is directly related to increased physical activity, and higher levels of physical activity are correlated with better quality of life. There is a relationship between increased sedentary behavior and low quality of life [17]. These relationships did not appear to differ between male and female genders [18].

Sedentary lifestyle refers to a pattern of daily life in which the individual does little to no exercise or movement on a daily basis. Therefore, it refers to the absence of movement on a daily basis with many hours of inactivity and immobility. According to the World Health Organization, a person is considered inactive when they do less than 150 min of physical activity per week [19]. Τhis lifestyle increases the widespread use of electronic devices, as well as work environments that demand extended periods of screen time and aggravate spinal discomfort, in the cervical region [19], through the avoidance of walking on a daily basis, leading to obesity [20]. A sedentary lifestyle has various effects on the musculoskeletal, the neurological system, and the psychological state [21]. There is a decrease in muscle mass and an increase in fat [22]. There are also metabolic changes such as sugar, pressure, and phlebitis due to poor venous return and blood circulation [23]. A study found that anxiety, poor posture, and reduced mobility exacerbate neck pain of office workers and decreased their daily quality of life [16]. Frequent complaints such as headaches [24] and high depression with a prevalence rate of 50% [25] in individuals with a sedentary lifestyle appeared. Johnston et al. [26] found a significant relationship between quality of life, neck pain, and daily use of mobile phones, tablets, computers, and television. Also, Sitthipornvorakul et al. [27] reported that reduced physical activity is associated with neck pain.

Only a limited number of studies have investigated gender differences in sedentary behavior and/or disability due to neck pain and/or quality of life among individuals with non-specific neck pain. In particular, women show higher levels of sedentary behavior at 33.8% worldwide, in contrast to men at 28.7% [28]. Sedentary women exercised for less than 30 min and less than 3 times a week and these women had a tendency towards obesity, depression, stroke, poor sleep quality, and increased sweating. Also, the women followed a sedentary behavior due to studying, driving, and working [29]. A large percentage of the male population spends more than 3 h a day using a computer, playing video games and driving a car. Furthermore, men had an exercise rate of 53%, while women only 48% and women with a rate of 34% are more inactive than men with a rate of nearly 29% [29].

The examination of gender differences and interrelationship among the pain-related disability, the sedentary lifestyle, and the quality of life within a single study population has not been previously conducted. Thus, the main aim of the present study was to assess gender differences in sedentary behavior, pain-related disability, and quality of life among individuals with non-specific neck pain and secondly to investigate the interrelationship of the three above measured variables with demographical characteristics as gender, age, and educational level. It is necessary to investigate if any differences exist in the above variables between two genders with chronic neck pain in order to develop detailed and specialized physiotherapy programs for these clinical populations.

## 2. Materials and Methods

### 2.1. Participants

We estimated an a priori minimum sample size using G*Power version 3.1.9.7. Based on an expected effect size of 0.80 and 90% power (a = 0.05, two-tailed) we aimed to recruit at least 68 participants (34 participants each group). Eighty participants (44 men and 36 women) with chronic non-specific neck pain participated in the present study. The convenience sample was recruited by word-of-mouth from private physiotherapy clinics from Athens by the second author. All the participants had previously visited an orthopedic physician where their neck region had been examined with clinical assessment and MRI.

The inclusion criteria for the sample were (a) aged 18–55 years, and (b) diagnosed with chronic non-specific neck pain (>90 days) [30]. Neck pain was defined as a score ≥5 on the Neck Disability Index (NDI) [30] for at least 3 months [31], (c) current management of non-specific neck pain through conservative treatment (i.e., medicine), (d) engagement in sedentary work for more than 4 h per 30 day [32], and (e) an ability to understand the Greek language.

Exclusion criteria included (a) history of neck injury and other pathologies of the cervical spine such as radiculopathy, (b) surgery in the head, face, cervical spine, upper or lower limbs, or cervical hernia, (c) degenerative disease of the spine, (d) presence of any rheumatological or cardiovascular disease, chronic neurological or psychiatric disorders, drug addiction, anemia, or diabetes, (e) use of analgesic, anti-inflammatory, or muscle relaxant drugs within the previous week, (f) pregnancy or breastfeeding, and (g) physiotherapy treatment within the past 3 months.

Participants signed the informed consent according to the Declaration of Helsinki. The study was approved by the Ethics Committee of the University of Peloponnese (Protocol Number: 12004/16-5-2025). The study was performed from May 2025 to July 2025.

### 2.2. Measurements

The participants completed specific, validated self-reported questionnaires that are used worldwide in patients with chronic neck pain [33].

(a) Neck Disability Index (NDI) [34,35]

The NDI is one of the most commonly used self-assessment tools for evaluating disability caused by neck pain. It includes 10 items that assess the impact of neck pain on daily activities. A 6-point scale (0–5) was used with higher scores indicating greater disability. The total score ranges from 0 to 50, and levels of disability are categorized as 0–4 (no disability), 5–14 (mild), 15–24 (moderate), 25–34 (severe), and 35–50 (complete disability). The NDI has been culturally adapted and validated for use in the Greek population [35]. Previous research has demonstrated its strong test–retest reliability (r = 0,30, *p* = 0,02) and good internal consistency (Cronbach’s α = 0.85–0.94) [34].

(b) EuroQol (EQ-5D-5L) [36]

The EQ-5D-5L is a standardized tool designed to measure general health status. It evaluates five key dimensions of health: mobility, self-care, usual activities (such as work, study, household tasks, and social or leisure activities), pain/discomfort, and anxiety/depression. Each dimension is rated across five levels of severity: no problems, slight problems, moderate problems, severe problems, and extreme problems or inability to function. The second component of the questionnaire is a visual analog scale (VAS) ranging from 0 (worst imaginable health) to 100 (best imaginable health), where participants rate their current overall health. The EQ-5D-5L is commonly employed in clinical and economic evaluations of healthcare interventions due to its high reliability, responsiveness, and strong validity. Moreover, it requires only a few minutes to complete [37]. The tool has also been translated and validated for use in the Greek population [38].

(c) International Physical Activity Questionnaire—Short Form (IPAQ-SF) [39]

The tool used to assess physical activity levels in the current study was the International Physical Activity Questionnaire—Short Form (IPAQ-SF) [39]. This questionnaire is widely used internationally to evaluate both physical activity and sedentary behavior in adults. The IPAQ is available in two main versions: the short form (IPAQ-SF) and the long form (IPAQ-LF). The present study used the short form version. The IPAQ-SF collects data on physical activity habits over the past 7 days across three main domains: work, transportation, and leisure-time activity. Respondents provide information on the frequency, duration, and intensity of their activities, including time spent sitting. It consists of 7 items. The explanations of the 7 IPAQ-SF items are:

1st item: During the last 7 days, in how many of them did you do vigorous physical activities such as digging, heavy weightlifting, running on an incline treadmill, fast running, aerobics, fast bicycling, fast swimming, singles tennis, or competitive sports (e.g., football, basketball, or volleyball)?

2nd item: On those days, how much time did you usually spend on vigorous physical activity?

3rd item: During the last 7 days, how many of them did you do moderate physical activities in such as carrying light loads (less than 10 kg), general house cleaning, gentle aerobic exercises, recreational cycling at a slow pace, or relaxed swimming? (Please do not include walking.)

4th item: On those days, how much time did you usually spend on moderate physical activity?

5th item: During the last 7 days, how many days did you walk for at least 10 continuous minutes?

6th item: On those days, how much time did you usually spend walking?

7th item: During the last 7 days, how much time did you spend sitting on a typical day? (This may include time spent sitting at home, at work, in a car or other transport, reading, socializing, resting in a chair, or watching television—but not including sleeping).

The IPAQ-SF has shown good internal consistency (Cronbach’s α = 0.70–0.90) and strong test–retest reliability (ICC = 0.65–0.91) [39]. It has been validated for the Greek population [40].

### 2.3. Procedure

Participants were informed about (a) the aim of the study, (b) the voluntary nature of participation, and (c) the confidentiality of their responses. Participants registered for the study and, having the inclusion criteria, were asked to sign an informed consent document. Questionnaires were administered to the participants before their first physiotherapy session in the physiotherapy clinics that they had attended for their chronic non-specific neck pain. The completion of the three questionnaires and a demographic sheet took an average of 15 min.

### 2.4. Statistical Analysis

The demographic data and the subscale scores of the three questionnaires were analyzed with descriptive statistics (i.e., means, standard deviations, and minimum and maximum number). Kolmogorov–Smirnoff tests were performed to assess normality.

Regarding the scoring of IPAG-SF, we calculated MET (Metabolic Energy Turnover, (MET minutes a week). MET minutes represent the amount of energy expended carrying out physical activity. To produce a continuous variable score from the IPAQ-SF (MET minutes a week), we considered walking to be 3.3 METS, moderate physical activity to be 4 METS, and vigorous physical activity to be 8 METS. There are three physical activity categories by scoring high, moderate, or low physical activity on the IPAQ-SF [39]. To calculate MET minutes per week, multiply the MET value given (walking = 3.3, moderate activity = 4, and vigorous activity = 8) by the minutes the activity was carried out and again by the number of days that that activity was undertaken. Adding the MET minutes, we achieved, in each category, (walking, moderate activity, and vigorous activity) production of the total MET minutes of physical activity a week [39].

Spearman’s rank correlation coefficient (Spearman r) was used to assess the relationships between the variables of disability due to neck pain, quality of life, and sedentary behavior. Also, we examined associations between demographical variables like gender, age, and educational level with the three measured variables of pain disability, quality of life, and sedentary behavior. Any differences between men and women on the three above variables were investigated by Kruskal–Wallis H tests. We calculated the effect size via r = z/√N (z: z value; N: observation number). In particular, we divided the z value to the square root of the observation number for to receive the effect size. The effect size r that is less than 0.3 means small effect. The effect size r between 0.3 and 0.5 means medium effect, and greater than 0.5 is the large 207 effect [41]. We also applied Bonferroni correction to multiple Spearman correlation analyses (thus changing alpha to 0.05/14 variables (disability, EQ-5D-5L Index Score and EQ-5D-5L VAS, total score of IPAQ-SF, 7 items of IPAQ-SF, gender, age, and education level) = 0.0035 in order to control the probability of making at least one type I error across the multiple correlations. Lastly, linear regressions analysis was performed to investigate the predictive ability of gender to neck disability, quality of life, and sedentary behavior. SPSS/PC for Windows, Version 28.00 was used for all statistical analyses.

## 3. Results

The 80 participants involved 44 men (55%) and 36 women (45%). The mean age of the total sample was 33.55 years (SD = 11.16, range 20–55 years). The average age of female participants was 36.25 years (SD = 9.13) and for males was 31.34 years (SD = 12.24). Participants reported engaging in sedentary work for an average of 6.04 h per day (SD = 2.13) over the past three months. Men reported a higher daily average in sedentary work (6.80 h, SD = 1.72) compared to women (5.11 h, SD = 2.24) and this difference was statistically significant (U = 434.50, *p* < 0.001).

According to the results of the IPAQ-SF regarding intense moderate exercise and walking in total MET/week for each gender, men had 2.3 MET*min/week which shows moderate physical exercise (at least 600 Met·min/week) and women had 1.86 MET*min/week on average of low to moderate physical activity.

All participants (100%) had visited an orthopedic surgeon due to neck pain and were undergoing conservative treatment (i.e., medicine). They were diagnosed with chronic non-specific neck pain, without any pain radiating to the upper limbs. The average duration since participants last experienced neck pain was 14.45 months (SD = 12.44). All participants were on their first visit to the physiotherapy clinic and they had not previously performed any physiotherapy treatment. Demographic characteristics of the total sample are presented in Table 1.

Table 2 presents the descriptive statistics of the total scores of the NDI, the EQ-5D-5L Health Survey, the IPAQ-SF for all the total sample (*n* = 80), and men (*n* = 44) and women (*n* = 36). Also, Table 2 reports the differences between the men and women. In particular, the variables: (a) disability due to pain (H = 13.96, *p* < 0.01), (b) quality of life (H = 16.14, *p* < 0.01), (c) IPAQ 2nd item (H = 3.37, *p* < 0.05), (d) IPAQ 4th item (H = 5.17, *p* < 0.05), and (e) IPAG 6th item (H = 11.24, *p* < 0.01) show a statistical difference between women and men. All the effect sizes are small, except for the variable disability due to pain, quality of life, and the 6th item of IPAQ-SF, which are medium (Table 2).

Table 3 presents the intercorrelations between demographical variables, i.e., sex, age, and educational level with pain disability, quality of life, and sedentary behavior with Bonferroni correction (*p* < 0.0035). In particular, a strong positive correlation was found between the NDI and EQ-5D Index Scores (r = 0.74, *p* = 0.002). Also, we found a low positive correlation between total score of IPAQ-SF with age (r= 0.30, *p* = 0.002). A negative correlation was found between NDI and the EQ-5D VAS (r = −0.65, *p* = 0.003). Furthermore, the EQ-5D Index was positively correlated with the IPAQ 7th item (r = 0.32, *p* = 0.001), and negatively correlated with the EQ-5D VAS score (r = −0.44, *p* = 0.001) (Table 3). Several inter-item correlations were also found within the IPAQ-SF 1st item was positively correlated with the 2nd item (r = 0.97, *p* = 0.001) while showing negative correlations with the 4th item (r = −0.31, *p* = 0.001) and the 5th item (r = −0.57, *p* = 0.001). IPAQ 2nd item showed negative correlations with the 4th item (r = −0.36, *p* = 0.001) and the 5th item (r = −0.60, *p* =0.001). The IPAQ 3rd item was positively correlated with the 4th item (r = 0.45, *p* = 0.001) Also, intercorrelations were found between the total score of IPAQ-SF and the 1st item (r = 0.83, *p* = 0.002), 2nd item (r = 0.83, *p* = 0.002), 3rd item (r = 0.39, *p* = 0.001), and 6th item (r = 0.56, *p* = 0.002) (Table 3).

Linear regression analyses showed that gender predicted the disability and quality of life, but not the total sedentary behavior. The multivariate model suggested that the gender explained a significant proportion of the variance in disability (R^2^ = 0.07, R^2^ adj = 0.06) and quality of life (R^2^ = 0.20, R^2^ adj = 0.19). This indicated that the gender accounted (a) for 7% of disability and the regression equation was significant (F-ratio = 6, 46%, *p* = 0.013, β = 0.27, t = 2,54 with *p* = 0.013) and (b) for 20% of quality of life and the regression equation was significant (F-ratio = 20.15%, *p* = 0.001, β = 0,45, t = 4.49 with *p* = 0.001). The final prediction formula for the disability is as follows: Υ = 3.10 + 6.60x and for quality of life, Υ = 5.13 + 4.68x.

## 4. Discussion

Prolonged daily use of electronic devices, sedentary office work, driving, and similar activities appeared to be associated with the onset of neck pain and reduction in quality of life. This is the first study that has investigated the relationship and gender differences in the three measured variables, i.e., disability, quality of life, and sedentary behavior, in one single study, in individuals with chronic neck pain. Our sample was relatively young, and among them, 64% did not have children, which showed that their disability may not be not high, their quality of life may be good, and they may have time to do physical exercise due to non-family obligations. The results of the present study showed that men with chronic neck pain had lower pain-related disability and better quality of life than women with chronic neck pain. Although men had a more sedentary behavior than women, a finding that is not confirmed by other studies, they do vigorous and moderate physical activity and walking for more time than women. Thus, men exercised more than women. These findings add to the existing knowledge.

Gender differences in the prevalence of neck pain have been reported in studies, which is proposed to be due to pain sensitivity and muscle anatomy between the genders [42]. Also, these differences in behavioral and psychological risk factors can influence musculoskeletal pain, e.g., females report a higher likelihood of poor posture and higher rates of co-morbid psychological distress [43]. The present results showed that gender predicted only neck disability and quality of life. Females generally experience worse outcomes, including higher disability levels and poorer quality of life compared to males. In particular, women experience neck disability more frequently than men, partly due to anatomical differences, higher rates of sedentary jobs, and conditions like headaches that are more prevalent in females. Anatomical reasons may be one reason for these gender differences. Women have smaller cervical vertebrae, less muscle strength, and ligament stiffness, which can lead to increased pain and lower tolerance for neck shear forces [10]. Also, hormonal fluctuations can also affect joint stability. Conditions that are more common in women, such as polymyalgia rheumatica, fibromyalgia, and rheumatoid arthritis, also increase neck pain risk [11].

Both genders are at increased risk for neck pain from sedentary lifestyles, but sedentary behaviors like long hours of driving and computer use contribute to neck pain differently across genders. Excessive screen time, prolonged sitting, and poor posture are key contributors. The everyday extended use of computers and phones are significant risk factors for both genders, with some studies showing a stronger association than just general prolonged sitting [44]. Another study found that women who worked in occupations with long hours of sedentary demands such as telephone operators, secretaries, and cashiers were more prone to developing chronic neck pain than men [45]. Results from another study showed that employees who sat for more than 4 h with very short rest breaks developed neck pain with women to be more prone to developing it [46]. Indeed, office workers who experience neck pain, stress, and reduced mobility exacerbate pain in the area. Neck pain occurs in office workers with an average of six hours of sitting per day [47]. A study of 29,496 volunteers found that work-related stress was associated with the occurrence of neck pain, with women being more susceptible [48]. In addition to general sedentary risks, a study on female students found that factors like prolonged self-study time, poor posture (e.g., flexed neck), and psychological distress were independent risk factors for neck pain. Indeed, among undergraduate healthcare students, the flexed neck posture >20 degrees the static duration posture >2 h and psychological distress were independent factors for neck pain in females [49].

In consistence with the present study, Yalcinkaya et al. [50] indicated that men with chronic neck pain demonstrated higher levels of physical activity and better quality of life than women with chronic neck pain. Similarly, Umeda et al. [51] examined gender differences in leisure-time physical activity among patients with neck pain. Their findings, in accordance with our results, showed that men dedicated more time to physical exercise during their free time compared to women, i.e., the male gender was more active in their daily lives compared to the female gender. Similarly, men reported higher levels of physical activity than women and performed more intense exercise than women [52]. Findings from Tsauo et al. [53] and Sitthipornvorakul et al. [54] indicated that reduced physical activity is associated with increased levels of neck pain. More specifically, Sitthipornvorakul et al. [54] reported that an increase in daily walking steps was associated with a reduced likelihood of developing neck pain. By reducing sedentary lifestyle and replacing it with daily exercise, there is a significant improvement in the levels of reported neck pain [54].

In the present study, a significant relationship was found between (a) neck-related disability and quality of life, (b) neck-related disability and pain, (c) items of IPAQ-SF and neck-related disability, and (d) hours spent sitting per day from IPAQ-SF and quality of life. Similarly, Luo et al. [55], using the NDI and the SF-36, found a strong negative relationship between the disability and quality of life measure. Pain intensity and an individual’s daily life significantly affect functional status and quality of life. However, the sample was not homogeneous and it was a convenience sample, as it was in the present study. Another study by MacDermid et al. [56] identified a significant association between the SF-36 and the NDI, confirming that the NDI is not only a valid and reliable tool for assessing neck disability, but also that it is strongly correlated with quality-of-life outcomes. A similar use of the IPAQ by Soysal et al. [57] showed that reduced physical activity is associated with decreased functional ability, poorer sleep quality, deteriorated psychosomatic condition, and lower quality of life. Disability and depression were positively and statistically significantly associated with physical activity levels and physical activity was associated with quality of life. As a result, these results are consistent with those of the present study. Citko et al. [58], also using IPAQ, indicated that factors such as sleep quality, caffeine intake, sedentary behavior, and lack of ergonomic workstations are positively associated with the NDI in hospital nursing staff with non-specific neck pain.

The clinical significance of the present results may increase awareness and attention in this population in order to decrease disability. Examining the relationship of the aforementioned variables may reduce pain symptoms and patients with chronic neck pain could have better quality of life. The present results highlighted the importance of physical activity and the reduction in sedentary behavior in maintaining good quality of life in patients with chronic neck pain. Such findings can help optimize individual prevention and treatment strategies for musculoskeletal pain since behavioral and psychological factors are potentially modifiable, unlike demographic characteristics.

### 4.1. Strengths and Limitations of the Study

Τhe large sample of patients with non-specific neck pain is one strength of the present study, which enhances the generalizability of the findings. The use of standardized assessment tools contributes to the validity of the data. The originality of the research topic is considered a significant strength, as it addresses an area with limited existing literature because no other study has, until now, examined disability due to neck pain, sedentary life, quality of life, and pain all together.

However, a limitation of the study is that the sample was heterogeneous in terms of demographic data such as age (our convenient sample was relatively young), educational level, having children, occupational status, total duration of sedentary behavior or physical activity, and acute pain characteristics (i.e., pain spreading, intensity, and impact). Another limitation is that we did not measure BMI of our sample and we exclusively used self-reported questionnaires. We have not used objective measurements of physical activity which measure movement, and other methods like pedometers, heart rate monitors, and direct observation. Accelerometers are a popular and non-invasive tool that can provide data on physical activity intensity, frequency, duration, and type. These devices capture the frequency, intensity, and duration of movement by analyzing the wearer’s acceleration. Also, we did not examine biological and hormonal factors associated with sedentary behavior to clarify their role in the development or persistence of neck pain. Lastly, we did not assess specific physiological or psychosocial mechanisms that are most plausible to explain gender differences in patients with chronic neck pain.

### 4.2. Future Recommendations

It is necessary to conduct studies with larger and more homogeneous samples, investigating whether sedentary work hours and reduced physical activity can predict the onset of neck pain. We recommend studies to explore the predicted ability of demographical data such as sex, age, BMI to pain disability, quality of life, and sedentary life. Similar research could be conducted on other chronic musculoskeletal conditions such as low back pain, assessing variables such as self-efficacy, depression, and attention during work-related tasks.

## 5. Conclusions

The study examined differences between men and women with non-specific chronic neck pain in terms of disability, quality of life, and sedentary behavior, as well as the relationship among these variables. Men reported better quality of life, higher levels of physical activity, increased time engaged in vigorous and moderate physical exercise, and lower levels of pain-related disability. Gender predicted neck disability and quality of life. These findings are in line with the other few studies. Future research should investigate whether neck pain can be predicted by sedentary work hours and reduced physical activity to determine the most effective strategies for managing chronic musculoskeletal pain, thus promoting better quality of life and improved occupational functioning.

## Figures and Tables

**Table 1:** Demographic characteristics of the total sample (*n* = 80).

Characteristics		Frequency	%
Education	Elementary	0	-
	Junior high school	4	5
	High school	49	61.3
	University	26	32.5
	Master’s degree	1	1.3
Marital status	Unmarried	10	12.5%
	Married	68	85%
	Divorced	2	2.5%
	Separated	-	
Number of children	0	51	63.7%
	1	8	10.%
	2	17	21.3%
	3	4	5.%
Profession	Office worker	26	32.5%
	Cashiers	14	17.5%
	Driver	13	16.3%
	Accountants	12	15%
	Bank employees	10	12.5%
	Restaurant manager	5	6.3%
Medicine for neck pain	Yes	11	13.8%
	No	69	86.3%
Last time of neck pain	1 week ago	25	31.3%
	2 weeks ago	15	18.8%
	3 weeks ago	20	25%
	1 months ago	7	8.8%
	2 months ago	13	16.3%
Neck pain affects everyday activities	Yes	79	98.8%
	No	1	1.2%
Neck pain affects your psychological state everyday	Yes	80	100%
Neck pain affects quality of sleep	Yes	68	85%
	No	12	15%
Doing physical activity in your free time	Yes	74	92.5%
	No	6	7.5%
Physical activity	Gym	32	40%
	Walking	38	47.5%
	Swimming	4	5%
Frequency of physical exercise	0 days/week	6	7.5%
	2 days/week	19	23.8%
	3 days/week	28	35%
	4 days/week	17	21.3%
	5 days/week	6	7.5%
	6 days/week	3	3.8%
	7 days/week	1	1.3%
Spending time (hour) on electronical devices in your free time	0 h/day	1	1.3%
	1 h/day	1	1.3%
	2 h/day	48	60%
	3 h/day	3	3.8%
	4 h/day	20	25%
	6 h/day	5	6.3%
	8 h/day	2	2.5%
Electronic device choice for spending time	Mobile	41	51.2%
	Television	15	18.8%
	Computer	9	11.3%
	Tablet	13	16.3%
	Nothing	2	2.5%

**Table 2:** Descriptive statistics and differences between gender in disability, quality of life, and physical activity.

	Total Sample (*n* = 80)	Men (*n* = 44 Men)	Women (*n* = 36)		
Questionnaire/Item	Mean ± SD (Min–Max)	Mean ± SD (Min–Max)	Mean ± SD (Min–Max)	Kruskal–Wallis H (Effect Size)	*p*
Neck Disability Index (NDI)	12.67± 11.94 (3.00–45.00)	9.70 ±8.37 (3–24)	16.30 ± 14.54 (6–45)	13.96 ** (0.42)	0.001
EQ-5D-5L Index Score	11.92 ± 5.17 (6.00–25.00)	9.82± 4.04 (6–16)	14.50 ± 5.29 (7–30)	16.14 ** (0.45)	0.001
EQ-5D-5L VAS	71.50 ± 13.64 (30.00–95.00)	74.36 ± 10.97 (60–95)	68.00 ± 15.78 (25–80)	1.78 (0.17)	0.109
Total Score IPAQ-SF	1666.75 ± 1519.85 (0.00–4817)	1884.22 ± 1533.52 (0.00–4817)	1489.27 ± 1503.11 (0–4697)	1.63 (0.16)	0.202
IPAQ-SF 1st item	2.10 ± 2.76 (0–6)	1.68 ± 2.65 (0–6)	2.61 ± 2.84 (0–6)	2.03 (0.16)	0.154
IPAQ-SF 2nd item	21.75 ± 29.02 (0–60)	28.33 ± 27.03 (0–60)	16.36 ± 30.38 (0–60)	3.37 * (0.16)	0.057
IPAQ-SF 3rd item	1.54 ± 2.40 (0–6)	1.61 ± 2.54 (0–6)	1.44 ± 2.25 (0–6)	0.11 (0.04)	0.738
IPAQ-SF 4th item	8.19 ± 17.72 (0–60)	22.92 ± 21.28 (0–60)	12.50 ± 10.03 (0–45)	5.17 * (0.25)	0.023
IPAQ-SF 5th item	2.00 ± 2.79 (0–7)	1.98 ± 2.62 (0–7)	2.03 ± 3.03 (0–7)	0.15 (0.04)	0.703
IPAQ-SF 6th item	16.04 ± 22.68 (0–60)	21.42 ± 22.19 (0–60)	11.64 ± 22.40 (0–60)	11.24 ** (0.37)	0.001
IPAQ-SF 7th item	3.91 ± 2.59 (0–8)	3.59 ± 2.87 (0–8)	4.31 ± 2.17 (0–8)	1.21 (0.12)	0.271

* *p* < 0.05, ** *p* < 0.01, NDI: Neck Disability Index, 1st item IPAQ: number of days with vigorous physical activity per week, 2nd item IPAQ: minutes per day of vigorous physical activity, 3rd item IPAQ: number of days with moderate physical activity per week, 4th item IPAQ: minutes per day of moderate physical activity, 5th item IPAQ: number of days walking at least 10 continuous minutes per day, 6th item IPAQ: minutes per day walking at least 10 continuous minutes, 7th item IPAQ: hours spent sitting per day.

**Table 3:** Correlations (Spearman r) between demographical data, disability due to pain, quality of life, and sedentary behavior.

Variables	Gender	Age	Educational Level	NDI	EuroQol	EuroQol VAS	Total Score IPAQ-SF	1st Item IPAQ	2nd Item IPAQ	3rd Item IPAQ	4th Item IPAQ	5th Item IPAQ	6th Item IPAQ	7th Item IPAQ
Gender	1													
Age	0.32	1												
Educational level	0.45 *	0.35 *	1					.						
NDI	0.42 *	0.68 *	0.14	1										
EuroQol	0.42 *	0.68 *	0.14	0.74 *	1									
EuroQol VAS	0.45 *	0.59 *	0.05	−0.65 *	−0.44 *	1								
Total score IPAQ-SF	0.14	0.30 *	0.30	0.22	0.22	0.39	1							
1st item IPAQ-SF	0.16	0.16	0.25	0.07	0.04	0.06	0.83 *	1						
2nd item IPAQ-SF	0.21	0.17	0.28	0.09	0.04	0.03	0.83 *	0.97 *	1					
3rd item IPAQ-SF	−0.04	0.13	0.01	−0.02	0.12	0.01	0.39 *	0.21	0.21	1				
4th item IPAQ-SF	−0.26	−0.09	−0.16	−0.22	−0.21	0.10	0.02	−0.31 *	−0.36 *	0.45 *	1			
5th item IPAQ-SF	−0.04	−0.08	−0.12	−0.10	−0.02	−0.03	−0.30	−0.57 *	−0.60 *	−0.19	0.18	1		
6th item IPAQ-SF	0.38 *	0.63 *	0.44 *	0.22	0.27	0.14	0.56 *	0.26	0.27	0.24	−0.06	0.05	1	
7th item IPAQ-SF	0.12	0.34 *	0.48 *	0.18	0.32 *	0.15	0.06	0.07	0.07	0.10	−0.04	−0.10	0.19	1

* Statistical significance with Bonferroni correction 0.0035. NDI: Neck Disability Index, 1st item IPAQ-SF: number of days with vigorous physical activity per week, 2nd item IPAQ: minutes per day of vigorous physical activity, 3rd item IPAQ: number of days with moderate physical activity per week, 4th item IPAQ: minutes per day of moderate physical activity, 5th item IPAQ: number of days walking at least 10 continuous minutes per day, 6th item IPAQ: minutes per day walking at least 10 continuous minutes, and 7th item IPAQ: hours spent sitting per day.

## Data Availability

The data presented in this study are available on request from the corresponding author due to ethical reasons.
